# The characterization of metabolites alterations in white adipose tissue of diabetic GK Rats after ileal transposition surgery by an untargeted metabolomics approach

**DOI:** 10.1080/21623945.2021.1926139

**Published:** 2021-05-11

**Authors:** Xiaorui Lyu, Kemin Yan, Weijie Chen, Yujie Wang, Huijuan Zhu, Hui Pan, Guole Lin, Linjie Wang, Hongbo Yang, Fengying Gong

**Affiliations:** a Peking Union Medical College Hospital, Chinese Academy of Medical Science and Peking Union Medical College, Beijing, China,; b Department of Surgery, Peking Union Medical College Hospital, Chinese Academy of Medical Science and Peking Union Medical College, Beijing, China; c Medical Research Center, Peking Union Medical College Hospital, Chinese Academy of Medical Science and Peking Union Medical College, Beijing, China

**Keywords:** Ileum Transposition (IT) surgery, White adipose tissue (WAT), untargeted metabolomics, Weight loss, Goto-Kakizaki (GK) rats

## Abstract

Dysfunction of adipose tissue could lead to insulin resistance, obesity and type 2 diabetes. Thus, our present study aimed to investigate metabolites alterations in white adipose tissue (WAT) of diabetic GK rats after IT surgery. Ten-week-old male diabetic GK rats were randomly subjected to IT and Sham-IT surgery. Six weeks later, the untargeted metabolomics in WAT of diabetic GK rats was performed. Differential metabolites were selected according to the coefficient of variation (CV) of quality control (QC) sample <30%, variable importance in the projection (VIP) >1 and P < 0.05. Then, the hierarchical clustering of differential metabolites was conducted and the KEGG database was used for metabolic pathway analysis. A total of 50 (in positive ion mode) and 68 (in negative ion mode) metabolites were identified as differential metabolites in WAT of diabetic GK rats between IT group and Sham-IT group, respectively. These differential metabolites were well clustered, which in descending order of the number of involved differential metabolites is ubiquinone and other terpenoid-quinone biosynthesis, AMPK signalling pathway, pantothenate and CoA biosynthesis, ferroptosis, vitamin digestion and absorption, glycerophospholipid metabolism, phenylalanine metabolism, steroid hormone biosynthesis, neuroactive ligand–receptor interaction, porphyrin and chlorophyll metabolism and bile secretion, and correlated with the parameters of body weight, food intake, WAT mass and glucose metabolism, which were significantly improved after IT surgery. The differential metabolites in WAT of diabetic GK rats were mainly related to the pathway of energy metabolism, and correlated with the improved phenotypes of diabetic GK rats after IT surgery.

## Introduction

Obesity is widely prevalent in the world. As a major risk factor of type 2 diabetes (T2DM), fatty liver, cardiovascular disease, hypertension, sleep apnoea syndrome and obesity have seriously decreased the quality of life and life expectancy [1,2]. Bariatric surgery is the most effective and durable treatment for obesity currently, most common procedures of which include gastric banding (GB), Roux-en-Y gastric bypass (RYGB) and sleeve gastrectomy (SG) [3–5]. Clinical evidence showed that bariatric surgery could effectively alleviate the above-mentioned multiple complications of obesity, as well as improve the quality of life and increase the life expectancy of patients [5]. Ileum transposition (IT) surgery is a promising bariatric surgery. It translocates a distal ileum segment to the proximal jejunum so that the food can stimulate the terminal ileum earlier, promoting the secretion of gastrointestinal hormones [6]. Recent studies in different animal models of diabetes/obesity found that IT surgery played an important role in weight loss, the reduction of food intake and fat mass, the improvement of glucose, lipid metabolism and insulin sensitivity [7]. Our previous study also found that IT surgery in diabetic GK rats significantly decreased the body weight, food intake and fat mass of rats, and evidently improved their glucose metabolism [8]. Our further investigation demonstrated that the browning of white adipose tissue (WAT) mediated by the activation of FGF21 signalling pathway may contribute to the beneficial effect of IT surgery [9]. However, the detailed mechanism of IT surgery still needs to be further clarified.

Metabolomics includes targeted and untargeted metabolomics, which can be used to detect the changes of small molecular metabolites (<1 kDa), so it is widely applied to the study of metabolic diseases [10,11]. The untargeted metabolomics is mainly applied to find biomarkers and explore mechanisms by searching for differential metabolites between experimental and control group as well as analysing the relationship between metabolic pathways and phenotypes. The targeted metabolomics is usually used to further verify the results of the untargeted metabolomics [12]. In recent years, metabolomics has been widely used to explore mechanisms of bariatric surgery. For instance, Wilhelmi’s study reported that morbidly obese patients after sleeve gastrectomy had significantly increased serum metabolites related to polyamine metabolism, which might be associated to the postoperative resolution of metabolic syndrome [13]. Besides, Yu’s study found that the faecal microbiome and metabolites among patients after RYGB or SG surgery were significantly changed, which might be closely related to postoperative weight loss and metabolic improvement [14]. However, there were few studies reporting the metabolites alterations in animals. Our previous study first identified 10 serum differential metabolites in Goto-Kakizaki (GK) rats after IT surgery, which was highlighted in the pathways of lipid metabolism, bile acid and incretin secretion [8].

It was universally acknowledged that WAT is widely distributed in the subcutaneous regions named subcutaneous adipose tissue (SCAT) and around the viscera regions named visceral adipose tissue (VAT). There are many differences between SCAT and VAT, including anatomical, cellular, molecular, physiological, clinical and prognostic differences [15]. The VAT is characterized by more cellular, vascular, innervated and contains a larger number of inflammatory and immune cells, lesser preadipocyte differentiating capacity and a greater percentage of large adipocytes when compared with SCAT. Besides, owing to there are more glucocorticoid and androgen receptors in VAT than in SCAT, adipocytes in VAT are more metabolically active and more sensitive to lipolysis as well as more insulin resistant than adipocytes in SCAT [16]. Therefore, the accumulation of VAT is associated with the development of cardiovascular disease, insulin resistance and T2DM [17]. However, it was still unclear the potential mechanism underlying the above beneficial effect of IT surgery as well as whether it is mediated by the improvement of the metabolites alterations in visceral WAT to a certain extent. Therefore, in this study, an untargeted metabolomics was used for the first time to detect the metabolites alterations in epididymal WAT of diabetic GK rats after IT surgery. Our results might provide a new evidence to deepen our understanding of the mechanism underlying the metabolic improvement of IT surgery.

## Materials and Methods

### 1. Ileal transposition (IT) surgery

Ten-week-old male diabetic GK rats (279.7 ± 4.0 g), purchased from the National Rodent Laboratory Animal Resources (Shanghai, China), were housed individually in standard cages at a 12-h dark/light cycle. Rats were fed with high fat diets (45% fat, H10045, Beijing HFK Bioscience Co. Ltd., Beijing, China). After being acclimated for 1 week, rats were randomly divided into IT group and Sham-IT group (n = 7 per group). IT surgery operation was performed as previously described [9]. The rats in the IT group were anaesthetized with 1.5–3% isoflurane, and then a segment of ileum was transposed to the proximal jejunum. Rats in the Sham-IT group were subjected to Sham-IT surgery without transposition. The body weight, food intake and fasting blood glucose levels of the rats were recorded. Two IT rats and one Sham-IT rat died of intestinal obstruction after surgery. Six weeks later, rats were anaesthetized with 2% isoflurane after a 12-h fasting, and blood samples were obtained by cardiac puncture. WAT, including epididymal adipose tissue, perirenal adipose tissue and inguinal subcutaneous adipose tissue, were obtained and weighted. The WAT mass percentage was calculated by the percentage of total body weight occupied by the total WAT mass. All animal experimental procedures were approved by the ethics committee of Peking Union Medical College Hospital (NO. XHDW-2017-017).

### 2. Metabolomic analysis

100 mg of epididymal WAT was homogenized in 500 μL lysis buffer (methanol: water = 1:1), placed at −20°C overnight, and then centrifuged at 12,000 rpm for 20 min at 4°C. The supernatant was freeze-dried and then dissolved with the lysis buffer, followed by centrifuged at 12,000 rpm for 20 min at 4°C. The supernatant was then sent to the metabolomic measurement using an ACQUITY UPLC I-Class (Waters, USA) coupled with a XevoG2-XS Qtof mass spectrometer (Waters, USA). Metabolites separation was performed using a Waters HSS T3 column (2.1 × 100 mm, 1.8 μm) at a flow rate of 0.45 mL/min. The column temperature was set at 45°C. The 0.1% formic acid in water and acetonitrile was used as mobile phases A and B, respectively. The gradient elution (v/v) of mobile phases B was set as follows (time, B%): (0 min, 1%), (1 min, 1%), (1.5, 20%), (11 min, 99%), (12 min, 98%) and (14 min, 1%). Mass spectra were acquired with capillary voltage at 2.5 kV in positive (ESI+) or negative (ESI−) ion electrospray modes. The ion source temperature was 100°C. Full data acquisition was performed by scanning from 50 to 1500 m/z in both ion modes.

The original data files were processed using the Progenesis QI software (Waters, USA). Further data processing was performed using the EZinfo software (Waters, USA). The orthogonal projection to latent structure-discriminate analysis (OPLS-DA) was used to filter irrelevant signals and explore the different metabolites between IT and Sham-IT groups, the quality of which was tested by cross validation. Moreover, differential metabolites were selected according to the coefficient of variation (CV) of quality control (QC) sample <30%, variable importance in the projection (VIP) >1, P < 0.05 in our study. Further comparison with KEGG database, HMDB database, and NIST database was conducted for the identification of metabolites. Hierarchical clustering and Heatmap mapping were carried out on the basis of the differential metabolites selected from the samples. Metabolic pathway analysis was performed by comparing different metabolites with the KEGG database through the IMPaLA tool.

### 3. Statistical analysis

Data were presented as mean ± standard deviation (SD). The t-test was used for data analysis, and the Mann–Whitney U-test was used when the data was not normally distributed. All of the statistical computations were run using SPSS version 22.0 (SPSS Inc., Chicago, IL, USA). The significance level was set at *P* < 0.05.

## Results

### 1. The PCA analysis of positive and negative ionization modes

**Figure 1.**
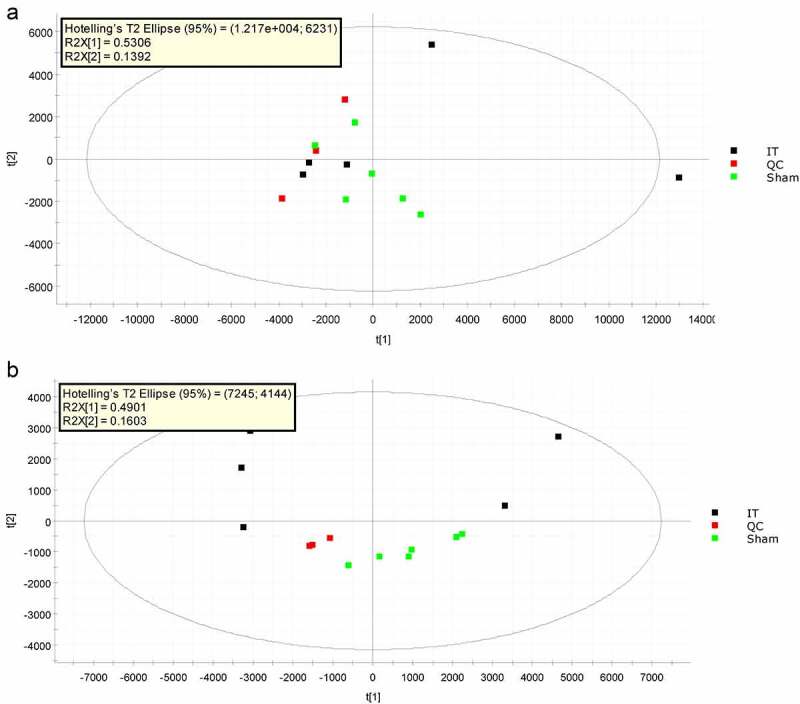
**PCA score plot of metabolic profiles of WAT sample of IT (n = 5) and Sham-IT (n = 6) rats in positive** (a) **and negative** (b) **ion modes**. 1 and 2 represented the first and the second principal component, corresponding to the x-axis and y-axis, respectively

As presented in the scatter plots of **Fig. S1**, our correlation analysis of QC samples showed that all correlation coefficients of data were greater than 0.80 in both positive and negative ion modes, suggesting good consistency. Besides, the peaks were narrow and large quantity in base peak chromatogram of the QC sample as detailed in **Fig. S2**, indicating the apparent effect of chromatographic separation. Moreover, the QC sample distribution was relatively concentrated in negative ion mode, demonstrating the reproducibility of the instrument was respectable. The QC sample distribution was relatively scattered in positive ion mode (Figure 1). However, the metabolites with CV<30% accounted for more than 80% of the total metabolites, thereby the instrument stability being excluded for dispersion of the sample distribution. In sum, the PCA analysis showed the good clustering quality.

### 2. The screening for differential metabolites in WAT of GK rats between IT and Sham-IT group

**Figure 2.**
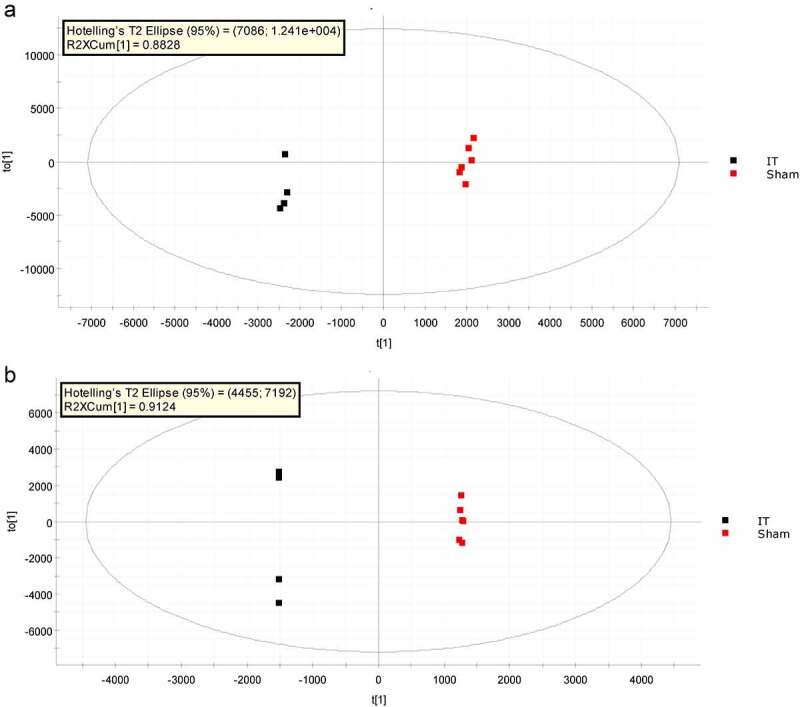
OPLS-DA analysis of metabolic profiles of WAT sample of IT and Sham-IT rats in positive (a) and negative (b) ion modes

**Figure 3.**
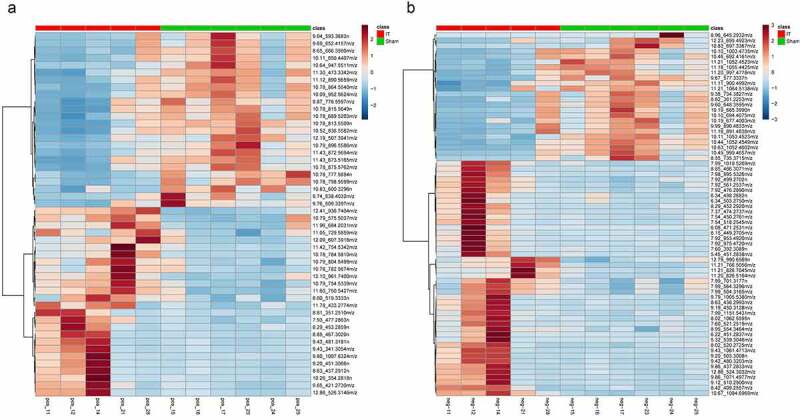
**Heatmap of differential metabolites of WAT sample of IT and Sham-IT rats in positive** (a) **and negative** (b) **ion modes**. The x-axis represented the name and classification of adipose samples, and Y-axis were the clustering results of different metabolites

As shown in Figure 2, R^2^X = 0.88, R^2^Y = 0.99, Q^2^ = 0.93 in the OPLS-DA score plot of the analysis in positive iron mode, while R^2^X = 0.91, R^2^Y = 0.99, Q^2^ = 0.90 in the OPLS-DA score plot of the analysis in negative iron mode. This was to say, there was no concern of over-fitting in the model, suggesting the samples were well grouped. The VIP value screening for metabolites was conducted through OPLS-DA model. The higher the VIP value of metabolites, the greater the contribution to the group. In our study, metabolites with VIP>1, P < 0.05 were selected as differential metabolites. A good effect of hierarchical clustering analysis of differential metabolites was shown in Figure 3. The characteristics of differential metabolites in WAT of diabetic GK rats between IT and Sham-IT in positive and negative iron mode were detailed in **Table S1** and **Table S2**, respectively. A total of 50 differential metabolites in positive ion mode were identified as shown in Figure 3a. Among them, 26 differential metabolites, such as LysoPE(0:0/18:2(9Z,12Z)), LysoPE(0:0/16:0) and LysoPC(18:2(9Z,12Z)), were elevated, while another 24 differential metabolites, such as PC(22:6(4Z,7Z,10Z,13Z,16Z,19Z)/20:2(11Z,14Z)), Lansiol and PS(P-20:0/22:4(7Z,10Z,13Z,16Z)), were decreased in IT group when compared with Sham-IT group. Besides, a total of 68 differential metabolites in negative ion mode were identified as detailed in Figure 3b. Among them, 44 differential metabolites, such as Simetride, 1VJB6VV6IA and LysoPE(0:0/20:5(5Z,8Z,11Z,14Z,17Z)), were up-regulated while 24 differential metabolites, such as PA(0:0/20:5(5Z,8Z,11Z,14Z,17Z)) and rolitetracycline were down-regulated in IT group in comparison with Sham-IT group .

### 3. KEGG pathway analysis of the differential metabolites in WAT of GK rats between IT and Sham-IT groups

**Figure 4.**
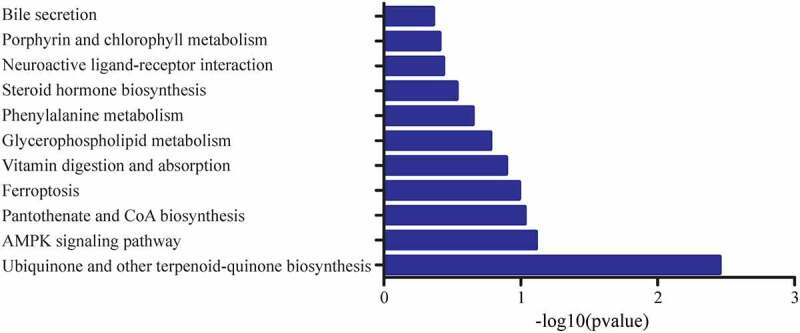
**The pathway analysis of metabolic profiles of WAT sample of IT and Sham-IT rats**. The x-axis represented the name and classification of adipose samples, and Y-axis were the clustering results of different metabolites

The metabolic pathways of related differential metabolites were analysed in comparison with the KEGG database. As demonstrated in Figure 4, the related pathways were ubiquinone and other terpenoid-quinone biosynthesis, AMPK signalling pathway, pantothenate and CoA biosynthesis, ferroptosis, vitamin digestion and absorption, glycerophospholipid metabolism, phenylalanine metabolism, steroid hormone biosynthesis, neuroactive ligand–receptor interaction, porphyrin and chlorophyll metabolism and bile secretion, which were presented in descending order according to the number of involved differential metabolites.

### 4. Potential relations between improved phenotypes of diabetic GK rats and differential metabolites in WAT

**Figure 5.**
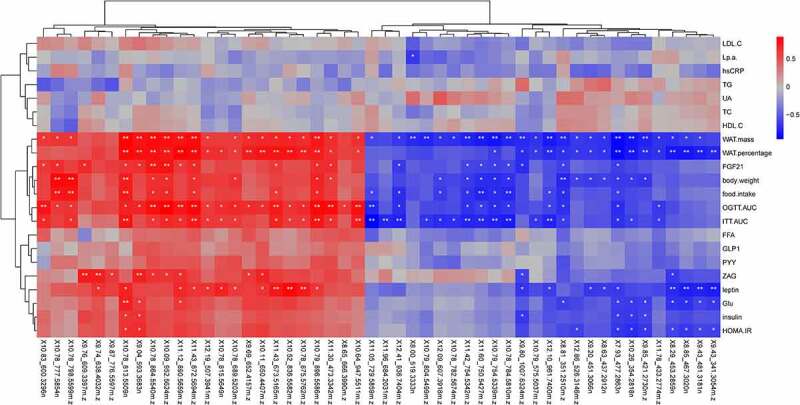
**Association map of spearman’s correlation analysis integrating phenotypes and differential metabolites in positive ion mode**. The x-axis represented the clustering results of different metabolites, and Y-axis were phenotypes. * *P* < 0.05 vs. the Sham-IT group, * * *P* < 0.01 vs. the Sham-IT group

**Figure 6.**
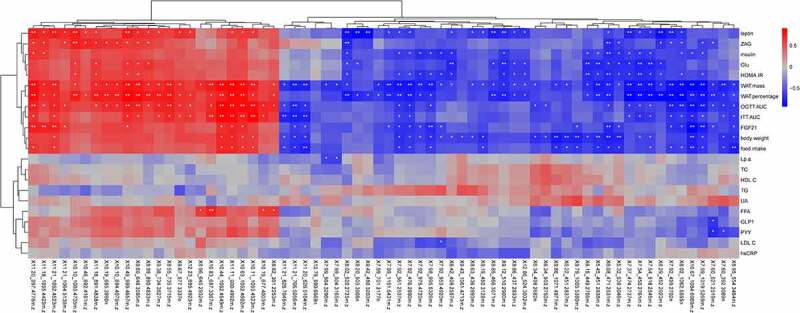
**Association map of spearman’s correlation analysis integrating phenotypes and differential metabolites in negative ion mode**. The x-axis represented the clustering results of different metabolites, and Y-axis were phenotypes. * *P* < 0.05 vs. the Sham-IT group, * * *P* < 0.01 vs. the Sham-IT group

To comprehensively analyse the relationship between the phenotypes of diabetic GK rats and differential metabolites found above, the Spearman’s correlation analysis was further performed in our study. As shown in correlation matrix of positive ion mode (Figure 5), seven differential metabolites, such as PS(O-16:0/20:0), PS(O-20:0/18:4(6Z,9Z,12Z,15Z)) and PS(18:0/20:3(8Z,11Z,14Z)), were positively, while another seven differential metabolites, such as PE(22:5(7Z,10Z,13Z,16Z,19Z)/P-16:0), PE(15:0/20:1(11Z)) and PC(20:2(11Z,14Z)/16:1(9Z)) were negatively correlated with body weight and food intake. Moreover, 16 differential metabolites such as PS(18:0/20:3(8Z,11Z,14Z)), PC(16:0/5:0(CHO)) and Ditekiren, were positively correlated, while 15 differential metabolites, such as LysoPE(0:0/18:2(9Z,12Z)), MG(0:0/18:2(9Z,12Z)/0:0) and CPA(18:0/0:0), were negatively correlated with total WAT mass and WAT percentage. In addition, PC(16:0/5:0(CHO)) and PS(18:0/20:3(8Z,11Z,14Z)) were positively correlated, while LysoPE(0:0/18:2(9Z,12Z)), MG(0:0/18:2(9Z,12Z)/0:0) and CPA(18:0/0:0) were negatively correlated with the blood glucose level, insulin level and HOMA-IR. As shown in correlation matrix of negative ion mode (Figure 6), there were five differential metabolites, including rolitetracycline, rose bengal (125I) sodium, macrocin and so on, were positively correlated with body weight and food intake, while 18 differential metabolites, including PE(16:0/18:1(Z))-15-isoLG lactam, methapyrilene, LysoPE(0:0/20:0) and so on, had negative correlation. Besides, there were 19 differential metabolites, including 1-Acetyl-3,27-dihydroxywitha-5, 24-dienolide 3-glucoside, GABUNINE, rose bengal (125I) sodium and so on, were positively correlated with WAT mass and WAT percentage, while 25 differential metabolites, including Myxalamid A, LysoPE(0:0/18:4(6Z,9Z,12Z,15Z)), LysoPE(0:0/20:5(5Z,8Z,11Z,14Z,17Z)) and so on, had negative correlation. Furthermore, there were four differential metabolites, including Kudzusaponin SA4, Vaspit, Travoprost and so on, were positively correlated with blood glucose level and HOMA-IR, while 13 differential metabolites, including (17alpha,23S)-17,23-Epoxy-29-hydroxy-27-norlanosta-1,8-diene-3,15,24-trione, Myxalamid A, LysoPE(0:0/18:4(6Z,9Z,12Z,15Z)) and so on, had negative correlation. These relations indicated that the improved phenotypes of diabetic GK rats after IT surgery might be involved in the differential metabolites of WAT in these rats.

## Discussion

Our previous study found that the body weight, food intake and fat mass were decreased in diabetic GK rats after IT surgery, along with the improvement of glucose metabolism. In this study, we found that these benefits of IT surgery might be associated to the metabolites alterations in WAT of diabetic GK rats between IT group and Sham-IT group, which were mainly involved in energy metabolism.

To the best of our knowledge, this is the first time that an untargeted metabolomics approach was performed to investigate the metabolites alterations in WAT of diabetic GK rats between IT and Sham-IT groups, which might be associated with the benefits of IT surgery. As a result, a total of 50 (in positive ion mode) and 68 (in negative ion mode) differential metabolites were identified from the epididymal WAT of diabetic GK rats between IT and sham-IT groups, respectively. These metabolites focused on the following eleven pathways, including ubiquinone and other terpenoid-quinone biosynthesis, AMPK signalling pathway, pantothenate and CoA biosynthesis, ferroptosis, vitamin digestion and absorption, glycerophospholipid metabolism, phenylalanine metabolism, steroid hormone biosynthesis, neuroactive ligand–receptor interaction, porphyrin and chlorophyll metabolism and bile secretion, which were mainly involved in energy metabolism. Our previous study identified 10 different metabolites from the serum samples of the same diabetic GK rats, which were mainly involved in bile acids, ergostane steroids, ether phosphor-ether lipids, glycerophospholipid, polyphenol metabolite, triglyceride and unsaturated fatty acid, which were closely related to lipid metabolism and incretin secretion [8]. Consistent with our results, Ashrafian’s study also reported that plasma bile acids, phenylalanine and energy-related metabolites were changed in Wistar rats after RYGB surgery [18]. Moreover, Jung’s study found that the level of ATP, ADP, AMP and phosphorylated AMPK increased in the liver of Sprague–Dawley rats after duodenal-jejunal bypass surgery, leading to the activated pathways of energy production, fatty acid and glucose decomposition [19]. In addition, Samczuk summarized the metabolites alterations in plasma, serum, urine and other tissue samples of patients or animals with obesity, T2DM and non-alcoholic fatty liver disease after various bariatric surgery and found that phenylalanine metabolism, pantothenate and CoA biosynthesis were the significantly activated pathways after bariatric surgery [20].

Our previous study found that IT surgery had obvious effects on weight loss and metabolic improvement in diabetic GK rats. The improved parameters after IT surgery focused on the following aspects, including WAT mass, WAT percentage, body weight and food intake, as well as the levels of blood glucose, insulin, HOMA-IR, FGF21 and leptin [8]. In this study, we further found that the differential metabolites in epididymal WAT of diabetic GK rats between IT group and Sham-IT group had a high correlation with the above-mentioned improved indicators in diabetic GK rats after IT surgery through spearman correlation analysis. Therefore, these results indicated that the differential metabolites of WAT were closely related to phenotypes of weight loss, fat loss, food intake reduction, and the improvement of glucose metabolism in diabetic GK rats after IT surgery.

Subsequently, the pathway analysis of differential metabolites was conducted in WAT of diabetic GK rats between IT and sham-IT group. AMPK, an evolutionarily conserved serine/threonine protein kinase, is a key factor regulating the energy metabolism in the body, which can inhibit lipid synthesis, glycogen synthesis, gluconeogenesis, protein synthesis and oxidative stress, as well as promote lipolysis, glycogen decomposition and maintain mitochondrial homoeostasis [21–24]. In our study, it was found that the differential metabolites were closely related to the AMPK pathway in diabetic GK rats between IT and Sham-IT group. Consistent with our results, a few of studies showed that the expression of AMPK in the muscle and adipose tissue of Sprague–Dawley rats after IT surgery were increased, which might be related to the increased glucose uptake and glycolysis [25–27]. In addition, Basso’s study reported that the phosphorylation level of AMPK was increased in the muscle tissue of obese Wistar rats after Glandular Gastrectomy surgery, with the improvement of liver and peripheral insulin sensitivity [28]. In brief, the above results indicated that the activated AMPK pathway might be associated with the improvement of the glucose metabolism and insulin sensitivity of WAT in diabetic GK rats after IT surgery.

Ubiquinone, also known as CoQ, is an endogenously synthesized lipid with redox activity. It is present in almost all kinds of cells, most of which are abundant in mitochondria [29]. CoQ played a key role in the electron transport chain on the mitochondrial membrane during aerobic respiration. The production of ATP depends on sufficient CoQ, and the lack of CoQ is closely related to the occurrence of various metabolic-related diseases [30,31]. In our study, the most differential metabolites in WAT are involved in the biosynthesis of ubiquinone and other terpene quinones. Kucharská’s study found that the expression of CoQ was significantly reduced in diabetic rats induced by streptozotocin (STZ) [32]. However, CoQ supplementation could reduce the levels of blood glucose, plasma HbA1c and lipid peroxidation in islet as well as improve glucose tolerance in STZ rats [33,34]. In addition, decreased expression of CoQ in serum and WAT was also observed in obese mouse models [35]. It was found that adding the reductive form of CoQ to food could significantly reduce body weight, inguinal WAT fat and inguinal WAT percentage of KKAy mice, which was a model of obesity and T2DM [36]. Therefore, the enrichment of differential metabolites in the pathway of the biosynthesis of ubiquinone and other terpene quinones might be related to the weight loss, fat loss and improvement of glucose metabolism in diabetic GK rats after IT surgery.

In addition, our first study found that the differential metabolites in WAT of diabetic GK rats after they were enriched in the pathway of the CoA synthesis. CoA is one of the important coenzymes for energy metabolism in the body. It plays an important role in the oxidative decomposition of fatty acids and glucose to form acetyl CoA, which participates in the tricarboxylic acid cycle in the mitochondria and provides a substrate for ATP synthesis [37]. However, the role of CoA in IT surgery had been not reported previously and more studies are needed to be done in the future.

Phenylalanine is one of the essential amino acids for humans. Studies found that the serum phenylalanine level of obese people was about twice as that of normal people, whereas the serum phenylalanine level was significantly reduced in obese people after LSG and RYGB surgery, which might be closely related to the change of insulin response [38,39]. Chlorophyll is a kind of natural pigment in plants which contains porphyrin structure. Li’s study found that early chlorophyll supplementation could inhibit weight gain and improve glucose tolerance in diet-induced obesity mice [40]. The above results indicated that IT surgery might play an important role in the weight and improve glucose tolerance in diabetic GK rats by promoting the metabolism of phenylalanine, porphyrin and chlorophyll in WAT. Bile acid, the main component of bile, is of great importance in the improvement of glucose and lipid metabolism after IT surgery [41,42]. In this study, it was found that some differential metabolites were enriched in the pathways related to bile secretion in WAT after IT surgery. However, the liver, instead of WAT, is the main organ that secretes bile [43]. There had been no studies that reported the activated pathway of bile secretion in WAT after IT surgery. Further studies are needed to confirm this finding.

However, this study has some potential limitations. First of all, the targeted metabolomics was not performed, which could further verify the differential metabolites identified by the untargeted metabolomics in our study. Second, we only analysed the correlation of the differential metabolites and the phenotypes of diabetic GK rats. Even so, the precise mechanism was still unknown and more studies were needed to explore it in the future.

## Conclusion

Our study first found a total of 50 (in positive ion mode) and 68 (in negative ion mode) differential metabolites in WAT of diabetic GK rats between IT group and Sham-IT group. These different metabolites were mainly involved in the pathways of AMPK, CoA and CoQ synthesis, which were mainly related to energy metabolism, and correlated with the beneficial effects of weight loss, food intake, fat loss and the improvement of glucose metabolism in diabetic GK rats after IT surgery. This study provided a new evidence to reveal the mechanism of IT surgery in the metabolic improvement. Further studies are needed to explore its precise mechanism, promoting IT surgery as a clinical treatment for obesity and diabetes in the future.

## Supplementary Material

Supplemental Material
