# Supplementary material for: The characterization of metabolites alterations in white adipose tissue of diabetic GK Rats after ileal transposition surgery by an untargeted metabolomics approach
Source: Adipocyte. 2021 May 11;10(1):275–84. doi: 10.1080/21623945.2021.1926139 (PMC8118414; doi:10.1080/21623945.2021.1926139)
Supplement: Supplemental Material [file KADI_A_1926139_SM6026.zip › Document.rtf]

Fig.S1 The Assessment of QC sample in positive (A) and negative (B) ion modes. The X axis and Y axis were log2 value for the strength value of metabolites in different experimental groups, respectively. 
Fig.S2 The base peak chromatogram of QC sample in positive (A) and negative (B) ion modes.
