# Supplementary figures and images for: The characterization of metabolites alterations in white adipose tissue of diabetic GK Rats after ileal transposition surgery by an untargeted metabolomics approach
Source: Adipocyte. 2021 May 11;10(1):275–84. doi: 10.1080/21623945.2021.1926139 (PMC8118414; doi:10.1080/21623945.2021.1926139)

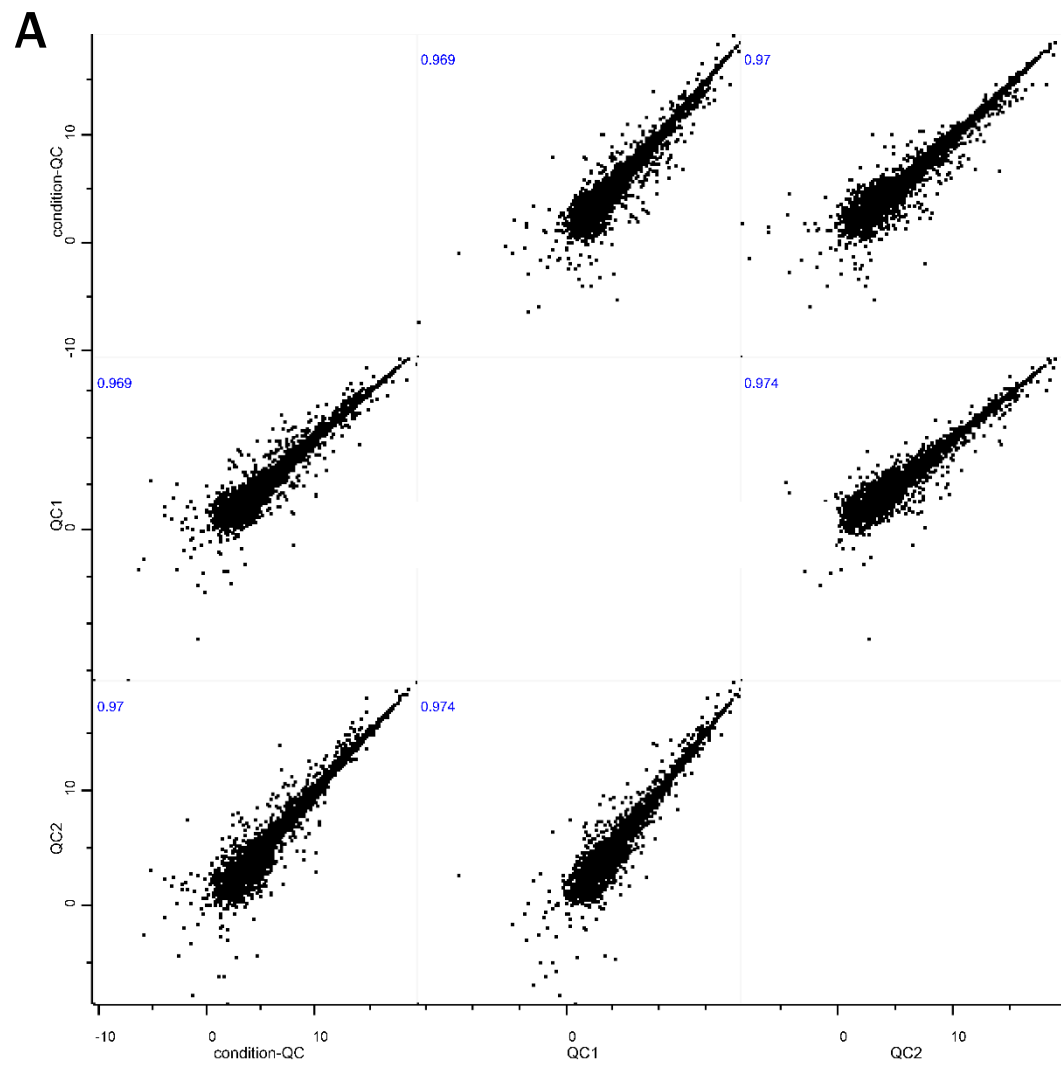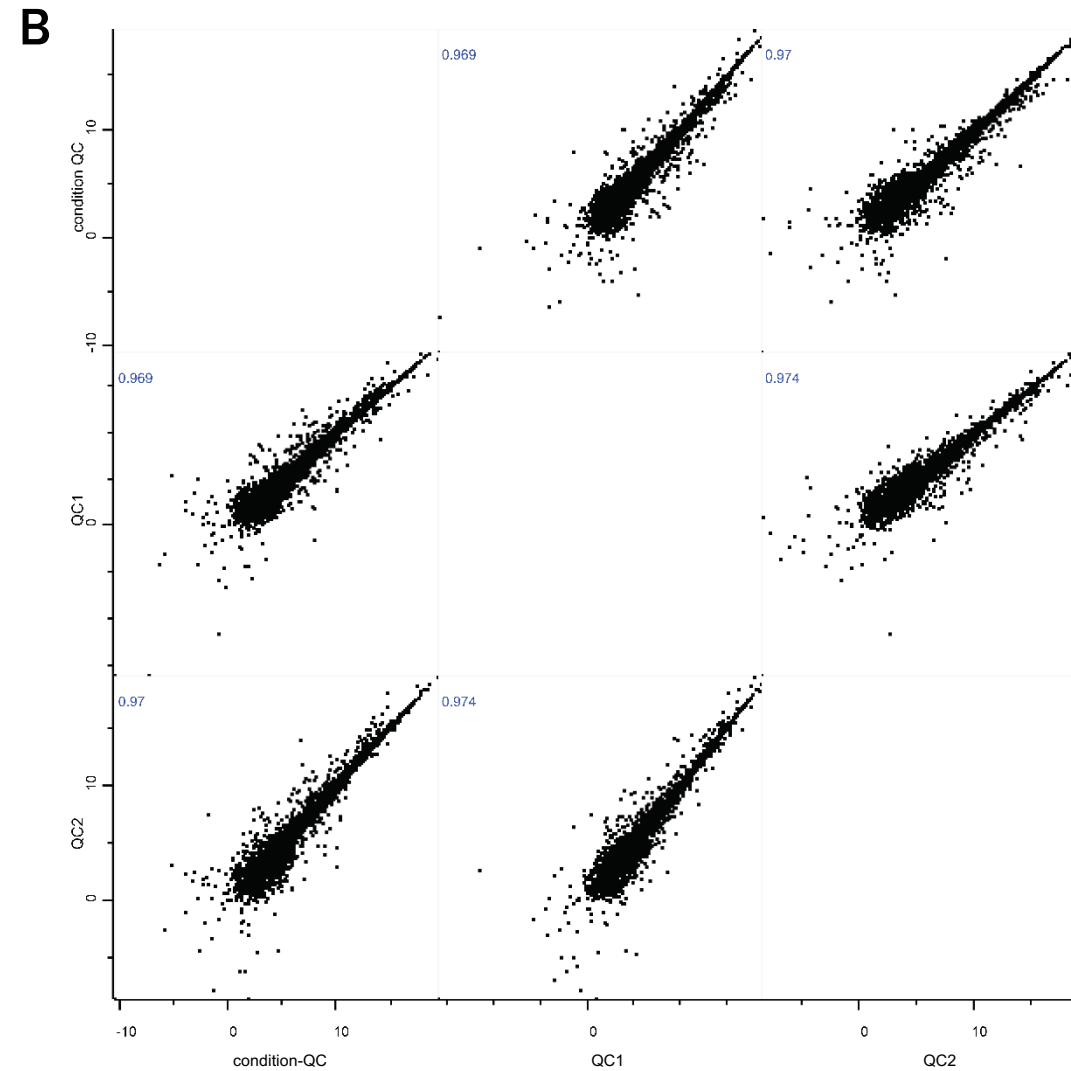

Supplement: Supplemental Material [file KADI_A_1926139_SM6026.zip › figs1.pdf]

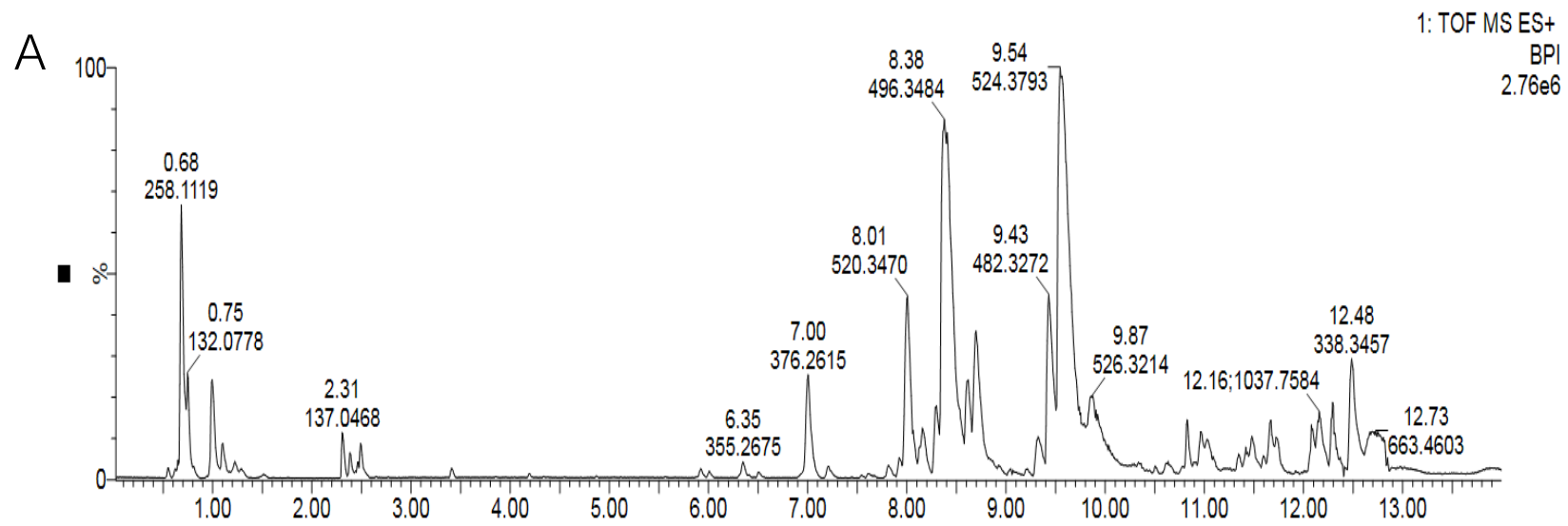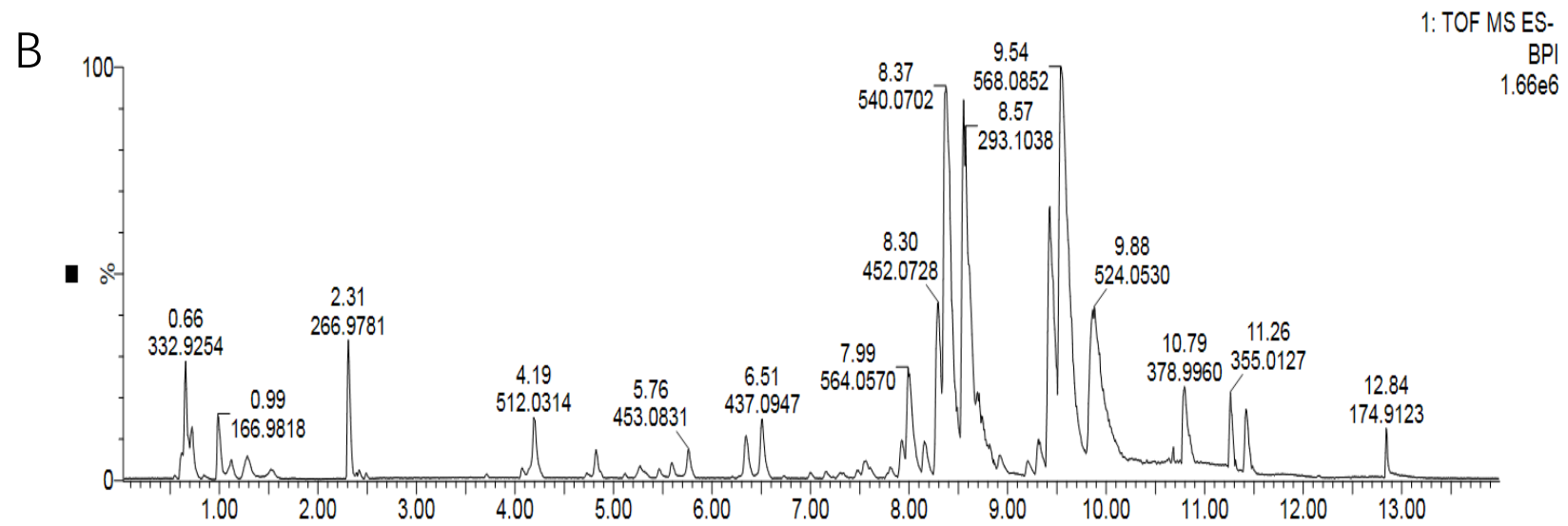

Supplement: Supplemental Material [file KADI_A_1926139_SM6026.zip › figs2.pdf]
